# Case of Pneumonia, Endocarditis, Pericarditis, and Meningitis

**Published:** 1840-10-24

**Authors:** W. W. Gerhard


MEDICAL EXAMINER.

DEVOTED TO MEDICINE, SURGERY, AND THE COLLATERAL SCIENCES.

No. 43.] PHILADELPHIA, SATURDAY, OCTOBER 24, 1840. [Vol. III.

*Case of Pneumonia, Endocarditis, Pericarditis, and Meningitis. Remarks on the pathology and treatment of Pneumonia. Extracted from a lecture delivered at the Philadelphia Hospital, by* W. W. Gerhard, M. D., 1840.

Abraham O———, aged 29, by trade a drayman, entered October 24, 1840. No anterior history. State, October 25.—Thin, dark complexion and hair; expression stupid; pupils natural; conjunctiva a little injected; flush of cheeks decided, (rounded form;) dilatation of nostrils; respiration forty-five, high; pulse one hundred and forty, easily compressed; skin moderately warm; (pulsations at wrist imperfectly formed;) delirium at night; subsultus; cough; no expectoration; tongue moist and clean; some fulness at hypochondrium.

*Percussion, anterior right side*—clear to third rib, thence flat to outer margin of nipple and centre of sternum. Impulse of heart diffused, tremulous, increased over body of ventricles; not much increased in loudness; second sound very deficient.

*Percussion, posterior right side*—lower half flat; respiration almost absent. Left side, respiration vesicular; bronchial rhonchi.

℞.—C. C. p. r. n.

Hyd. c. Mit.

P. Ipecac. *aa.* gr. ½ } q. h. sec.

Inf. Senegæ. et Eupatorii.

*Oct.* 26.—On evening of 24th had ℥vj. of blood taken by cups from shoulders posteriorly; do. on evening of 15th, from right side.

Has taken almost no food; delirium almost constant; had C. C. No. ij. to nucha last night without benefit. Has taken eighteen pills without amendment; face deeply flushed, of a bronzed tint; great dilatation of nostrils; pupils rather contracted; decubitus dorsal; restless; agitated; some subsultus; doubtful answers, articulation distinct; tongue clean and moist; bowels not open since 24th; respiration fifty-six, noisy at distance; pulse one hundred and forty to one hundred and fifty, very feeble; skin rather dry and warm; loose, noisy cough; no expectoration; heart's impulse strong, very much diffused; double bruit de scie near the valves, extending into aorta; the proper sounds of heart lost, except towards right side; doubtful creaking; complete flatness over præcordial region, from nipple to third rib and margin of sternum.

*Respiration, posterior right side*—Crepitant rhonchus very intense; intense bronchial respiration throughout whole lung. *Respiration anteriorly* feeble; *percussion* perfectly flat.

Treatment continued, with addition of one-quarter grain of digitalis to each dose.

Died during night of 26th.

*Oct.* 28. *Examination thirty-six hours after death.*—Very little emaciation; bluish tint of whole surface; ecchymosis slight; no infiltration.

*Right Lung.* Slight adhesions between the pleura pulmonalis and costalis; large deposite of soft yellow lymph upon pleura costalis; lung coated with yellow lymph, soft, easily detached, of considerable thickness, in the form of a slight film; pleura appears reddened from injection of vessels beneath; whole lung very heavy, sinking readily in water, especially lower and middle lobes.

*Upper Lobe* intensely red, containing bloody serum; still crepitant, (in first stage.)

*Lower Lobe* hardened, granulated, not floating in water, breaks readily under pressure, (second stage.)

*Middle Lobe* solid and highly granulated; pours out a slightly yellowish liquid; breaks up easily.

*Bronchial mucous membrane* reddened and thickened.

*Left Lung.* Effusion to the amount of ℥v. or ℥vi. in pleura; lung perfectly healthy.

*Heart* six inches in length, four and a half in breadth.

*Pericardium, externally*, much injected, and very vascular; adheres slightly to heart; is easily detached; contains two or three ounces of yellow serum, slightly turbid. *Anterior* part of it covered over its whole surface with a reticulated coating of lymph; slightly thicker at origin of vessels than elsewhere; yellowish and soft, of same apparent date as that on pleuræ. The same appearance extending over whole exterior of heart. Veins greatly distended. Injection but slight upon the surface of the heart.

*Aorta* contains a dark coagulum extending into ventricles; partially covered with a semi-organised membrane, irregularly injected. A finger passed readily into ventricle.

*Semi-lunar valves of Aorta* much injected, covered with a delicate pellicle of lymph. Valves thinned, but not ulcerated.

*Left Ventricle.* Lining membrane reddened, and finely injected in spots; free from deposites of lymph, the remainder being simply reddened.

*Semi-lunar valves of Pulmonary Artery* reddened, thinned, inflamed, with slight deposites of lymph.

*Mitral valve of Left Ventricle* reddened, but not ulcerated.

*Liver* enlarged, engorged with blood, fatty.

*Stomach* contains dark bilious matter, somewhat contracted. Mucous membrane of a dark gray colour; no bright injection; irregular streaks of softening; consistence elsewhere good.

*Spleen* much enlarged; twice its natural size; of a pale brown colour.

*Kidneys* healthy. *Small Intestines* do. *Mesenteric Glands* small. *Peyer's* healthy. Some venous distention of lower portion of intestines.

*Large Intestines* healthy; cicatrizations, with irregular contractions and thickening from previous dysentery.

*Brain.* Dura mater adherent in portions to skull. Slight effusion of turbid serum beneath it. A thin coating of nearly transparent serum spread over whole surface of brain. Membranes at base decidedly injected.

Moderate distension of veins.

Brain of good consistence, perhaps a little harder than natural. Ventricles contain but little serum.

*Remarks.*—This case illustrates a variety of pneumonia which is extremely prevalent in certain years. It is the highly inflammatory variety in which the inflammation is not confined to the lungs, but extends to other tissues, and notably to the serous membranes of the circulating system and the brain. That is, the inflammatory diathesis is developed by the same morbid impression which acts more especially upon the parenchyma of the lung, or the inflammation of other viscera are secondary in point of time to that of the lungs, and depend upon the disorder of the whole system which follows the local point of inflammation. In a majority of cases, and the present instance is one of them, the inflammatory action appears to occur simultaneously in various tissues of the body, and the pneumonia is only one part of the general disorder. The disease is rather an inflammatory condition of the vascular system, than a local affection, and the first pathological alteration which takes place in the body is probably dependant upon the condition of the blood itself. This fluid is in such cases highly charged with fibrine, which is thrown out upon the inflamed surfaces in the form of false membranes, and is, as every one knows, excessively abundant in the blood which is drawn from a vein. My own clinical observations had long ago convinced me of this fact, and a series of experiments of a chemical nature, which have recently been instituted by Dr. Andral, have proved the same fact by another process of observation, thus confirming the accuracy of the former mode of observation.

Pneumonia of a highly sthenic condition is therefore to be regarded as a general inflammatory disease which is much more extended in its action than would appear from the portion of the tissue of the lung which is affected, and the blood participates largely in the disorder. The diagnosis of this variety of pneumonia may be made partly from the local signs of disease of the different organs, and partly from the evident disorder of the blood, as shown by the physical condition of this fluid, and by the condition of the circulation. When we recollect that the heart and brain are the organs which are most apt to suffer, we naturally investigate their condition, and if we find the signs of meningitis, and of pericarditis, or endocarditis, we have sufficient evidence of the nature of the disorder. The symptoms of meningitis are delirium of an acute kind, and other signs of cerebral inflammation; those of endocarditis are the confused action of the heart, and the alteration of its sounds, which are either diminished in loudness, or the second sound becomes very feeble, and the first roughened. There is also a bruit de scie, or rough double sound, very different from the normal double sound of the heart, if the inflammation should extend into the aorta, as it did in the present instance. These signs serve us in the advanced stages of the disorder more than the state of the pulse, for as the action of the heart becomes oppressed, the blood is no longer driven with its accustomed freedom into the arteries, and the pulse may therefore fail; but at first the pulse is strong, full, highly characteristic of a strongly developed inflammatory diathesis.

The prognosis in these cases is dependent upon the gravity and the duration of the lesions; if they are very extensive, and the disease has lasted long enough for a coagulum to form in the heart, and for the inflammation of the capillaries of the brain to be confirmed, the probabilities of recovery are very slight; but at the commencement of the disorder, the disease may be arrested by a decided antiphlogistic treatment, and the prognosis is but a little more grave than in ordinary cases of pneumonia.

The treatment in the present case was wanting during the whole period of the disorder when it would have been of much avail. The patient was totally neglected up to the time of his admission into the hospital. We were then in a great degree paralyzed, and could do but little. The main reliance was constant local depletion, with a mercurial practice. If ptyalism should supervene, you may sometimes succeed even in this stage of the disorder; but although the antiplastic powers of mercury are very decided, they are of course uncertain if the development of the disease has reached an extreme point, and the disorganization of important organs extended as far as in the case now treated of. In the early stages of the acute and sthenic forms, the treatment is very simple, and, in general, is very decided in its effects.

It is in this variety that the antiphlogistic method of practice is most successful, and it is mainly conducted by copious blood-letting, which may, in some cases, be repeated several times, according to the method which has of late years been revived by Dr. Bouillaud, and by antimony. The blood letting, at the very commencement of the disorder, may occasionally succeed in cutting it short, but in the larger majority of cases it cannot be arrested, although it may be rendered more mild and many of its symptoms may be removed. For the rules relative to blood letting in this affection, I would refer you to my lectures on this subject, published during the winter and summer course of this year, and to the various works of Dr. Bouillaud, although I do not approve of the extremely large depletion which he advises. The antimonial practice, that is, the use of antimonials in high or contra-stimulant doses, is appropriately fitted to this form of the disorder, but only to a particular stage of it, that is, before the blood has become fixed in the tissue of the lungs, or coagulated in the heart; in the case now examined, this period was obviously past. For the rules governing the use of this remedy, I must also refer to the same source as to those respecting the prescription of blood letting.

